# Pulmonary edema following central nervous system lesions induced by a non- mouse-adapted EV71 strain in neonatal BALB/c mice

**DOI:** 10.1186/s12985-017-0911-5

**Published:** 2017-12-28

**Authors:** Yuefei Jin, Chao Zhang, Rongguang Zhang, Jingchao Ren, Shuaiyin Chen, Meili Sui, Guangyuan Zhou, Dejian Dang, Jiehui Zhu, Huifen Feng, Yuanlin Xi, Haiyan Yang, Guangcai Duan

**Affiliations:** 1 Department of Epidemiology, College of Public Health, Zhengzhou University, No.100 Kexue Avenue, Zhengzhou, Henan 450001 China; 2 Henan Collaborative Innovation Center of Molecular Diagnosis and Laboratory Medicine, Xinxiang, People’s Republic of China; 3 Department of Epidemiology, College of Public Health, Xinxiang Medical University, Xinxiang, People’s Republic of China; 4 Department of Infectious Diseases, The Fifth Affiliated Hospital of Zhengzhou University, Zhengzhou, Henan People’s Republic of China

**Keywords:** Enterovirus 71, Hand foot and mouth disease, Pulmonary edema, Central nervous system

## Abstract

**Background:**

Enterovirus (EV) infection has been a serious health issue in Asia-Pacific region. It has been indicated that the occurrence of fatal hand foot and mouth disease (HFMD) cases following EV71 infection is mainly attributed to pulmonary edema. However, the development of pulmonary disorders after EV71 infection remains largely unknown. To establish an EV71-infected animal model and further explore the underlying association of central nervous system (CNS) invasion with pulmonary edema, we isolated a clinical source EV71 strain (ZZ1350) from a severe case in Henan Province.

**Methods:**

We evaluated the cytotoxicity of ZZ1350 strain and the susceptibility in 3-day-old BALB/c mice with intraperitoneal, intracerebral and intramuscular inoculation. Various histopathological and immunohistochemical techniques were applied to determine the target organs or tissue damage after infection. Correlation analysis was used to identify the relationship between CNS injury and pulmonary disorders.

**Results:**

Our experimental results suggested that ZZ1350 (C4 subtype) had high cytotoxicity against African green monkey kidney (Vero) cells and human rhabdomyosarcoma (RD) cells and neonatal BALB/c mice were highly susceptible to the infection with ZZ1350 through three different inoculation routes (2 × 10^6^ pfu/mouse) exhibiting severe neurological and respiratory symptoms that were similar to clinical observation. Viral replication was found in brain, spinal cord, skeletal muscle, lung, spleen, liver, heart of infected mice and these sections also showed histopathological changes. We found that brain histology score was positive correlated with lung histology score in total experimental mice and mice under the three inoculation routes (*P* < 0.05). At the same time, there were positive correlations between spinal cord score and lung score in total experimental mice and mice with intracerebral inoculation (*P* < 0.05).

**Conclusions:**

ZZ1350 strain is effective to establish animal model of EV71 infection with severe neurological and respiratory symptoms. The development of pulmonary disorders after EV71 infection is associated with severity of CNS damage.

**Electronic supplementary material:**

The online version of this article (doi: 10.1186/s12985-017-0911-5) contains supplementary material, which is available to authorized users.

## Background

Enterovirus (EV) genus of the Picornaviridae family containing EV71 and coxsackievirus A16 (CA16) are the causative agents of hand foot and mouth disease (HFMD). Recently, outbreak of HFMD caused by EV71 infection have been a serious threat to infants and children in East and Southeast Asia. EV71 was first isolated in California, United States in 1967 and then had been recognized as a cause of epidemics of HFMD in Japan since 1973 [1, 2]. It has been acknowledged that EV71 is responsible for HFMD contributing to severe neurological complications and even fatalities in infants and young children worldwide [3, 4]. Generally, EV71 infection is a typically mild, self-limiting childhood disorder. However, a handful of cases can develop into devastating clinical outcomes presenting as either acute flaccid paralysis, aseptic meningitis, brainstem encephalitis, encephalomyelitis or pulmonary edema, once EV71 invades into central nervous system (CNS) [4, 5]. Although, current studies involving animal models of EV71 infection are well established to explore the pathological process [6, 7], the development of EV71-related pulmonary edema still remains largely unknown. Therefore, it is extremely urgent to reveal the pathogenesis of fatal EV71-induced pulmonary diseases and make great efforts to identify novel therapies for severe cases.

In this study, we separated an EV71 strain from a nonfatal case with CNS involvement in Henan Province. Unlike other animal models, 3-day-old mice were directly infected with clinical source virus purified and cultured in African green monkey kidney (Vero) cells and human rhabdomyosarcoma (RD) cells, not a mouse adapted strain. Additionally, mice in our experiments were inoculated via three infection routes and diverse outcomes were assessed. A variety of histopathological and immunohistochemical techniques were applied to demonstrate that whether EV71-induced CNS lesions are associated with occurrence of pulmonary edema.

## Methods

### Virus and cells

EV71 strain ZZ1350 was isolated from a nonfatal case with CNS involvement in Children’s Hospital of Zhengzhou (Zhengzhou, Henan, China). Viral growth was performed in human rhabdomyosarcoma (RD) cells, and virus purification was conducted as previously described [8]. RD cells and African green monkey kidney (Vero) cells (ATCC CCL-81) were cultured in Dulbecco’s modified Eagle’s medium (Gibco Company, New York, USA) containing 10% fetal bovine serum (FBS) (Gibco Company, New York, USA). Enrichment of the virus was performed using Dulbecco’s modified Eagle’s medium (Gibco Company, New York, USA) containing 2% fetal bovine serum (FBS) (Gibco Company, New York, USA). The virus stocks were stored at −80°C. TCID_50_ were determined by plaque assay using RD cells [9], and viral titers were expressed as PFU per ml. Working stocks in the present study contain 10^8^ PFU per ml.

### Viral RNA isolation and quantitative real-time PCR (qRT-PCR)

Sixty hour after infection, RD cells were collected and viral RNA was extracted using Hipure viral RNA kit (Magen Biotech Co. Ltd., Guangzhou, China). The first-strand cDNA was synthesized using a HiScript® Q RT SuperMix for qPCR (+g DNA wiper) (Vazyme Biotech Co. Ltd., Nanjing, China) according to the manufacturer’s protocols. EV, EV71, CA16 genes were assessed using SensoQuest Labcycler PCR system (SensoQuest Co. Ltd., Goettingen, Germany) with a 2 × Taq Master Mix (Vazyme Biotech Co. Ltd., Nanjing, China) for PAGE and gene-specific primers (Sangon Biotech Co. Ltd., Shanghai, China). The primer sequences of the viral genes were listed in Table 1 and the lengths of qRT-PCR products were 116 bp, 226 bp and 208 bp respectively.

**Table 1:** Primers used for RT-PCR

Target gene	Forward primer (5′ to 3′)	Reverse primer (5′ to 3′)	Source
EV	TCCGGCCCCTGAATGCGGCTAATCC	ACACGGACACCCAAAGTAGTCGGTCC	PrimerBank
EV71	GCAGCCCAAAAGAACTTCAC	ATTTCAGCAGCTTGGAGTGC	PrimerBank
CA16	ATTGGTGCTCCCACTACAGC	TCAGTGTTGGCAGCTGTAGG	PrimerBank

### Analysis of VP1 sequence and comparison

Briefly, the fragment of VP1 genome was amplified by qRT-PCR using the specific primers [Forward primer (5′ to 3′)**:** GCAGCCCAAAAGAACTTCAC), Reverse primer (5′ to 3′): ATTTCAGCAGCTTGGAGTGC]. Sequencing of PCR amplification products was conducted by Shanghai Sagan Corporation. Evolutionary tree based on VP1 genome was performed using MEGA software.

### Cell viability assay

The Vero cells (2 × 104/well) were seeded in 96-well plates in a total volume of 200 ul for 24 h, and then were infected with EV71 at an MOI of 0, 0.4, 1 and 5 for 24 h. We investigated the effect of EV71 infection on the viability of Vero cells using a MTT assay kit (Vazyme Biotech Co. Ltd., Nanjing, China) at 24 h post infection (hpi).

### Animals

Specific pathogen free 3-day-old BALB/c mice were obtained from the Medical Animal Center in Zhengzhou University, Henan, China, and raised in individual ventilation cage (IVC) system in the Medical Animal Center located in the College of Public Health of Zhengzhou University (temperature 20–24°C, humidity 40%–60%, lights on 8 a.m.-8 p.m.).

Mice were intracerebrally (*n* = 7), intramuscularly (*n* = 6), intraperitoneally (*n* = 6) inoculated with ZZ1350 strain (2 × 10^6^ PFU), and observed twice daily for 15 days for clinical symptoms and mortality, and body weight of mice was recorded every 2 days. Clinical scores of mice were defined as follows: 0, healthy; 1, reduced mobility; 2, ruffled hair, hunchbacked, or ataxia; 3, weight loss; 4, limb weakness and 5, dying or death. Normal mice (*n* = 5) were used as control.

### Histology

Forty eight mice with different inoculation routes (*n* = 15 for intracerebral inoculation; *n* = 12 for intramuscular inoculation; *n* = 11 for intraperitoneal inoculation) and normal controls (*n* = 10) were euthanized by isoflurane inhalation at 7 days post infection (dpi). The slices of brain, spinal cord, skeletal muscle, lung, spleen, small intestine, liver, heart and skin of mice were fixed in 4% paraformaldehyde at 4 °C overnight. After fixation, paraffin-embedded tissues were cut into sections of 5 μm in thickness and stained with haematoxylin and eosin (H&E). Paraffin-embedded lung tissues were also stained with Masson’s Trichrome and Periodic Acid-Schiff (PAS). Alveolar space of the lung was assessed through determination of the mean linear intercepts, L_m_ calculated based on 10 randomly selected fields in each section at 100× magnification with two crossed test lines [10]. Histology score of brain, spinal cord and lung was evaluated as reported in a previous publication [11], which was quantified by a person blinded to the treatment groups.

### Determination of viral titers

The tissue samples (brain, spinal cord, skeletal muscle, lung, spleen, liver, heart) (*n* = 5 for each inoculation route and normal control) harvested from euthanized animals were homogenized in sterile phosphate-buffered saline (PBS) (10% [wt/vol]), disrupted by three freeze-thaw cycles, and centrifuged with 8000×g per min for 5 min at 4°C. Viral titers in the supernatants of tissue lysates was determined by plaque assay and expressed as TCID_50_ per gram.

### Immunohistochemical staining

Viral VP1 protein expression in brain, spinal cord, skeletal muscle and was examined with immunohistochemical (IHC) staining. Briefly, brain, spinal cord and muscle tissue were first blocked with goat serum for 30 min at room temperature, and then were incubated with rabbit anti-VP1 specific polyclonal antibody (GeneTex, Inc., San Antonio, USA 1:500 dilution) over night at 4 °C, followed by incubation with HRP conjugated anti-rabbit secondary antibody at a concentration of 1:3000 at 37 °C for 30 min. Astrocytes in brain were identified by glial fibrillary acidic protein (GFAP). The brain section was incubated overnight at 4 °C with primary anti-mouse monoclonal GFAP (Millipore, CA, USA 1:500 dilution), followed by incubation with HRP conjugated anti-rabbit secondary antibody at a concentration of 1:3000 at 37 °C for 30 min. Positive IHC staining was presented as brown staining in cytoplasm and captured under a light microscope.

### Electron microscopy

The infected RD cells were fixed in 2.5% gluteraldehyde in 0.1 M sodium cacodylate buffer (pH = 7.4) for 1 h. The cells were rinsed in sodium cacodylate and those in petri dishes were scraped and spun down in 2% agar. All samples were fixed in 1% osmium tetroxide for 1 h, stained en masse in 2% uranyl acetate in maleate buffer (pH = 5.2) for a further hour, rinsed and dehydrated in an ethanol series, and infiltrated with resin (Embed812 EMS) and baked over night at 60 uC. Hardened blocks were cut using a Leica Ultra Cut UCT. 60 nm sections were collected on formver/carbon-coated grids and contrast stained using 2% uranyl acetate and lead citrate. Samples were viewed on an FEI Tecnai G2 Spirit Biotwin TEM at 200 kV. Images were taken using Morada CCD and iTEM software (Olympus Optical Co. Ltd., Tokyo, Japan).

### Immunofluorescence staining

The Vero cells (5 × 105/well) were seeded in confocal dish in a total volume of 1.2 ml medium for 24 h, and then infected with EV71 at an MOI of 0, 0.4, 1 and 5 for 24 h. Cells were immediately fixed with 4% paraformaldehyde for 30 min, followed by permeablilization in FACS buffer containing 0.1% saponin for 15 min at room temperature. Cells were then incubated with rabbit anti-EV71 polyclonal antibody (GeneTex, Inc., San Antonio, USA) at 1:1000 dilution at 4°C for 30 min, followed by staining with Cy3 conjugated anti-rabbit secondary antibody (Beyotime Biotech Co. Ltd., Shanghai, China) at a dilution of 1:200 at 37 °C for 60 min and protected from light. At last, DAPI was added with 1:2000 dilution at 4°C for 5 min. Images were captured using a Leica TCS-SP8 confocal microscope (Leica Microsystem, Wetzlar, Germany). EV71 positive area (%) was evaluated using Image-Pro Plus 6.0 software.

### Statistical analysis

All experiments were repeated twice. Data is presented as mean ± standard error of the mean (SEM). SPSS21.0 (IBM, Chicago, IL, USA) was used for statistical analysis. Data comparison was carried out by one-way ANOVAs followed by Dunnett’s post-test and two-tailed Student’s t-test. Clinical scores of experimental mice were analyzed by one-way ANOVA. Survival rates of infected mice were analyzed with Log-rank test. Correlation was evaluated using the Pearson’s correlation test. A two-tailed *P* value <0.05 was considered statistically significant.

## Results

### Isolation and cytotoxicity analysis of ZZ1350 strain

**Fig. 1:**
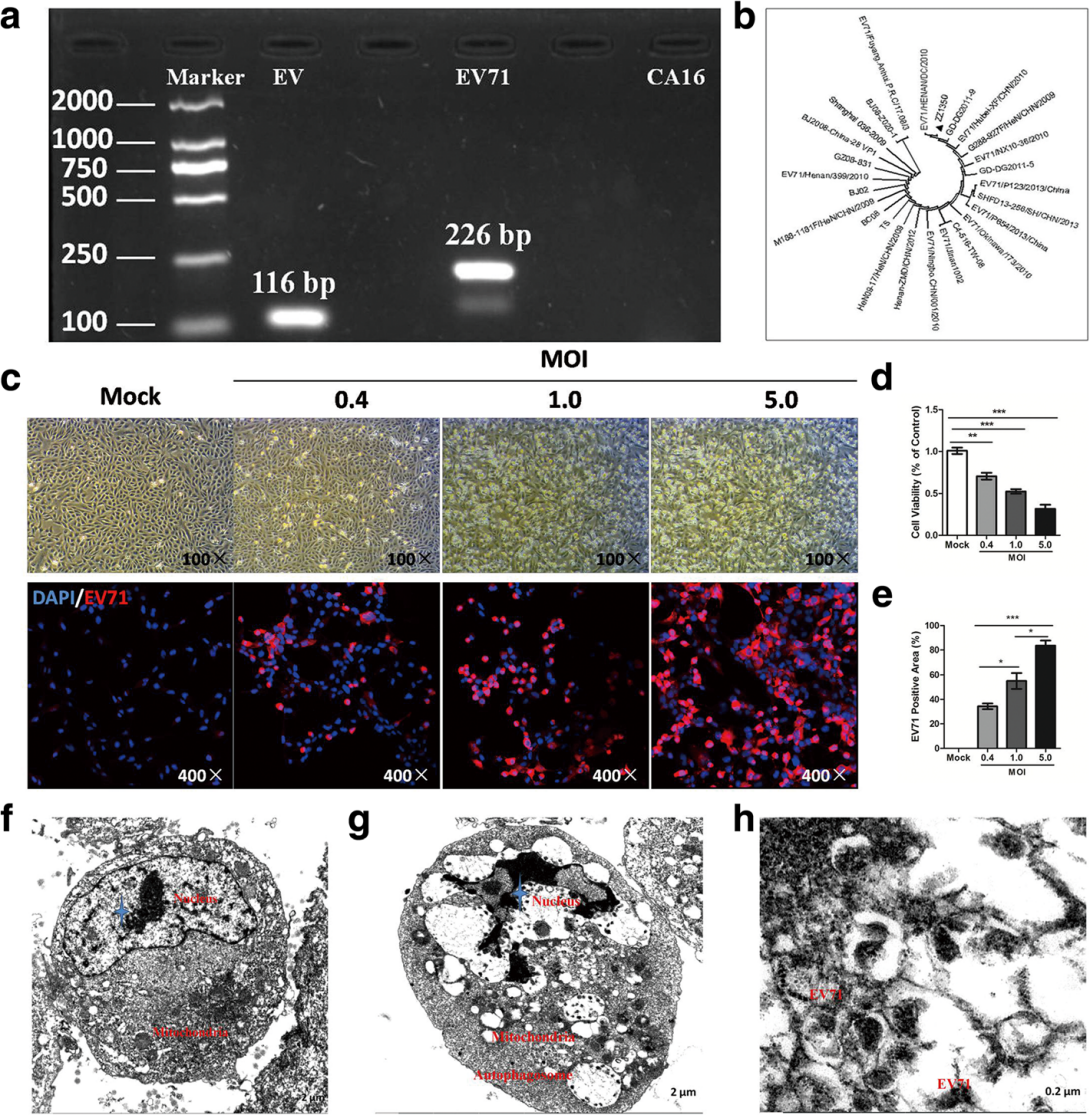
ZZ1350 infection led to high cytotoxicity against Vero cells and RD cells. Viral RNA in ZZ1350-infected RD cells was extracted and identified by qRT-PCR (**a**). Evolutionary tree based on VP1 genome (**b**) was performed using MEGA software. CPEs and EV71 distribution (**c**) in Vero cells induced by different doses of ZZ1350 were imaged via light microscopy (amplification: 100×) or confocal microscopy (amplification: 400×) at 24 hpi. The viabilities of Vero cells (**d**) following virus infection were determined using the MTT assay. EV71 positive area (%) (**e**) was evaluated using Image-Pro Plus 6.0 software. Cell pellets of normal (**f**), infected RD cells (**g**, **h**) were fixed and investigated with electron microscopy. Data are expressed as mean ± SEM. * *P* < 0.05, ** *P* < 0.01, *** *P* < 0.001 (*n* = 3)

ZZ1350 infection induced pronounced cytopathic effect (CPE) in RD cells at 60 hpi (Additional file 1: Figure S1). To define whether it was belonged to EV71 virus, Vero cells were collected and viral RNA was extracted. Our experimental results from qRT-PCR showed that EV and EV71 are all positive, but not CA16 (Fig. 1a). Evolutionary tree (Fig. 1b) based on VP1 genome sequence (Additional file 2) showed that ZZ1350 (GenBank KY886010) is similar to a reported strain in Henan, China, attributed to C4 subtype. ZZ1350 displayed pronounced cytotoxicity against Vero cells and RD cells at 24 hpi. ZZ1350 infection caused cytopathic effect (Fig. 1c) and cell variability reduction (Fig. 1d) in Vero cells with a dose-dependent relationship. EV71 positive expression area (Fig. 1c and e) was elevating with the increase in the dose of infection. Electronmicroscope was used to determine the cytotoxicity against RD cells at 24 hpi. Compared to control cells (Fig. 1f), ZZ1350 infection led to cytoplasmic vacuolization, nuclear shrinkage and autophagy in RD cells (Fig. 1g). EV71 particles were also found in cytoplasmic of RD cell (Fig. 1h). These results suggested that ZZ1350 strain (C4 subtype) exhibited a high cytotoxicity in vitro.

### Lethality of ZZ1350 strain in neonatal mice

**Fig. 2:**
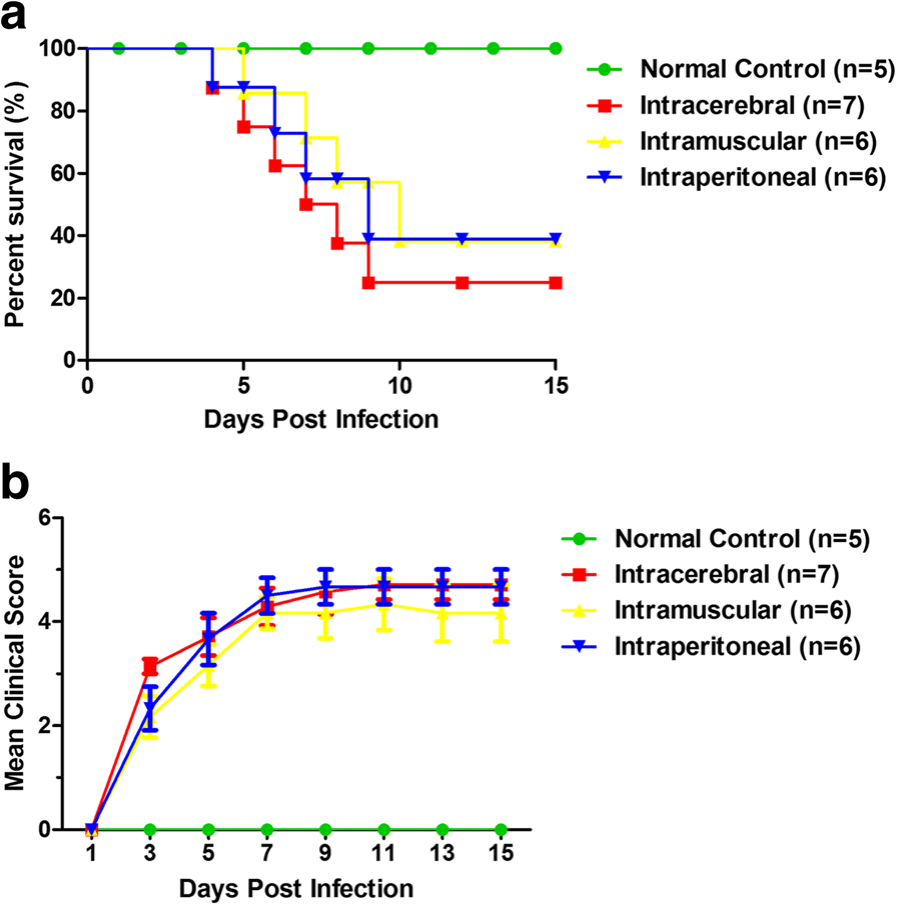
Viral virulence of ZZ1350 in neonatal mice. 3-day-old BALB/c mice were inoculated with 2 × 10^6^ PFU ZZ1350 strain via intracerebral (*n* = 7), intramuscular (*n* = 6) and intraperitoneal injection (*n* = 6). Survival rates (**a**) and mean clinical scores (**b**) of mice were monitored and recorded every 2 days after infection. *n* = 5 for normal controls; Data are expressed as mean ± SEM

To investigate potential differences in survival rates of neonatal mice following ZZ1350 infection, 3-day-old BALB/c mice were inoculated with 2 × 10^6^ PFU ZZ1350 strain via intracerebral, intramuscular and intraperitoneal injection. Clinical scores and the survival rates of neonatal mice infected with viruses were recorded. As shown in Fig. 2a, intracerebrally (14.3% survival; *P* < 0.05), intramuscularly (33.3% survival; *P* < 0.05) and intraperitoneally inoculated mice (16.7% survival; *P* < 0.05) exhibited shorter survival time compared to normal controls (100% survival). These findings suggested that ZZ1350 strain exhibited high level of lethality in neonatal mice with these three different routes. The mean clinical scores (Fig. 2b) of ZZ1350 strain infected mice with intracerebral, intramuscular and intraperitoneal injection was reached the similar level at 7 dpi, which was higher than that in normal controls (*P* < 0.05). These results indicated that the inoculation routes had limited influence on the outcomes of infected mice.

### ZZ1350 infection induced severe symptoms in neonatal mice

**Fig. 3:**
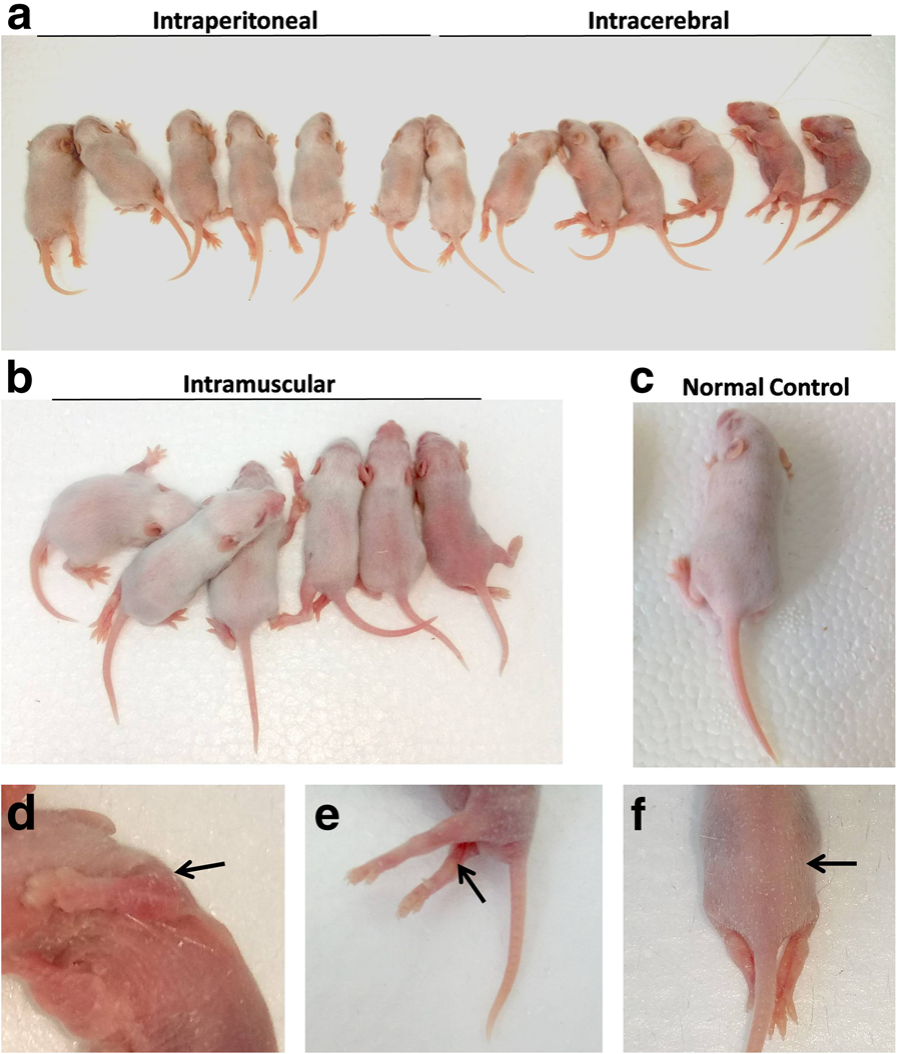
Clinical presentation in neonatal mice following ZZ1350 infection. Different inoculation routes including intraperitoneal (**a**), intracerebral (**a**) and intramuscular (**b**) injection were performed in the present study. Normal mice (**c**) were regarded as control. Severe infected mice exhibited flaccid paralysis, either in the forelimbs (**d**), hind limbs (**e**) or both; and hairless lesions (**f**) were also investigated at 5 dpi. Black arrows showed haemorrhagic lesions in the hind limbs or forelimbs

Mice inoculated with ZZ1350 strain via three routes emerged various severe symptoms respectively. Relative to normal controls (Fig. 3c), infected mice with different inoculation routes (Fig. 3a and b) presented with severe signs including reduced mobility, ataxia, weight loss (See Additional file 3: Figure S2), spastic limb paresis and/or paralysis (fore-limbs, hind-limbs, or both; see Additional file 4: Figure S3) or dying status. It was worth noting that severely affected animals presented progressive limb paralysis, haemorrhagic lesions on the joint of limb due to vasculitis, and hairless lesions on the back (Fig. 3d-f). Additionally, some animals even have signs of respiratory distress including tachypnea and gasping. Taken together, our findings suggested that mice infected with ZZ1350 exhibited severe neurological and respiratory symptoms.

### Viral replication of infected neonatal mice

**Fig. 4:**
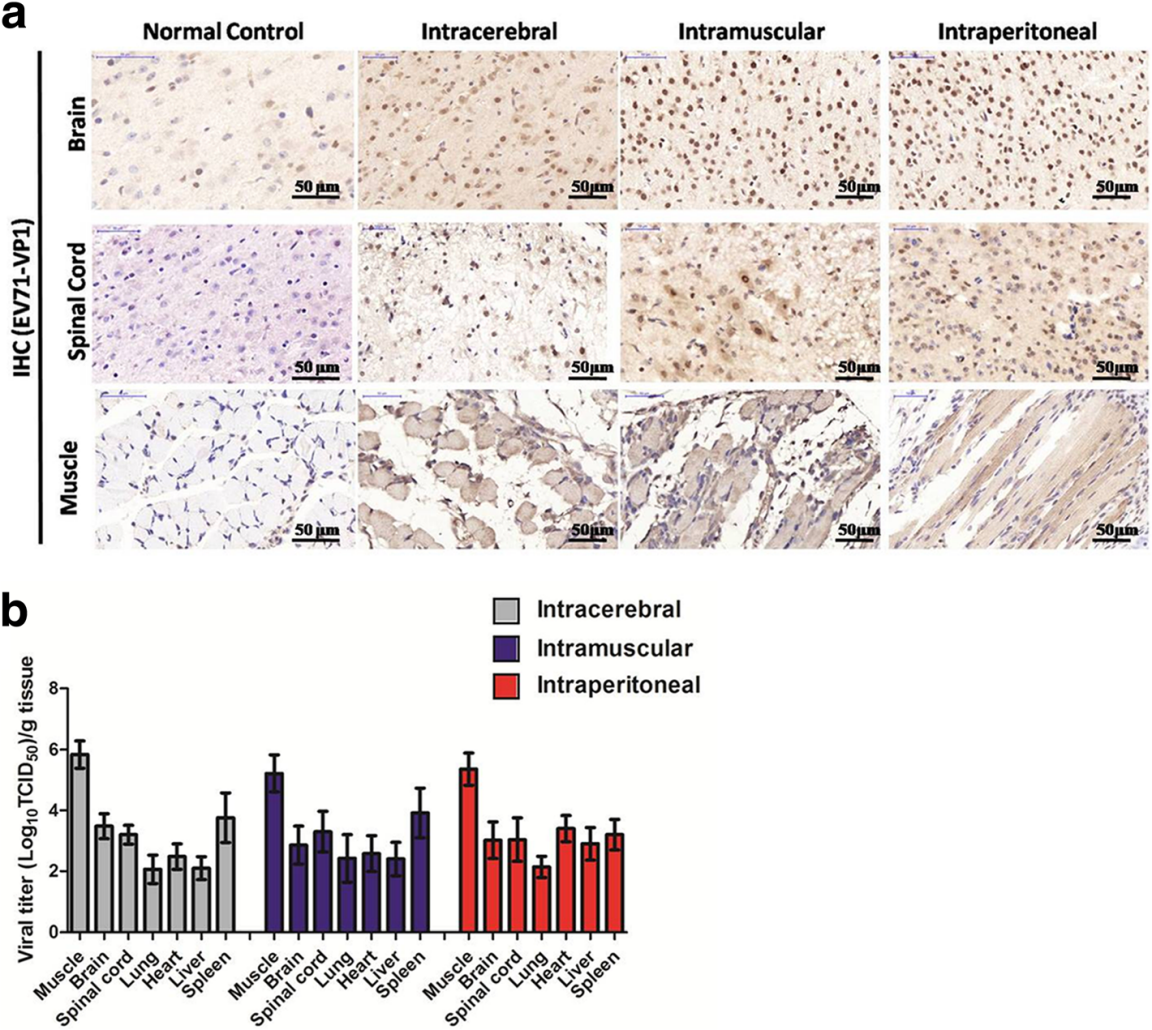
Viral replication in tissues of ZZ1350-infected mice. The IHC expression and localization of VP1 represented as brown staining in cytoplasm (**a**) were shown for tissues from the brains, spinal cords, skeletal muscle of ZZ1350-infected mice. Viral titers in brain, spinal cord, skeletal muscle, lung, spleen, liver, heart (**b**) was expressed as log_10_ TCID50 per gram (*n* = 5 for each inoculation route and normal control)

The expression of EV71 VP1 protein and distribution of virus particles in infected animals were determined by IHC staining and plaque assay. As shown in Fig. 4a, mice inoculated through any of the three routes showed a higher density and stronger intensity of VP1 positive signals in neurons located in the brain and spinal cord or skeletal muscle of mice with three inoculation routes relative to normal controls. These results suggested that virus could replicate in CNS section and skeletal muscle of infected animals, and further caused extremely similar disease progress. Next, viral titers in brain, spinal cord, skeletal muscle, lung, spleen, liver, heart of infected mice were evaluated at 7 dpi. As shown in Fig. 4b, relative to other organs, high level of viral titers was determined in skeletal muscle, brain, spinal cord and spleen of infected animals. Virus titers in lung and liver were lower than that in other tissues. These results indicated that ZZ1350 strain was a CNS- and muscle-tropic virus.

### Histology of various tissues of ZZ1350-infected mice

**Fig. 5:**
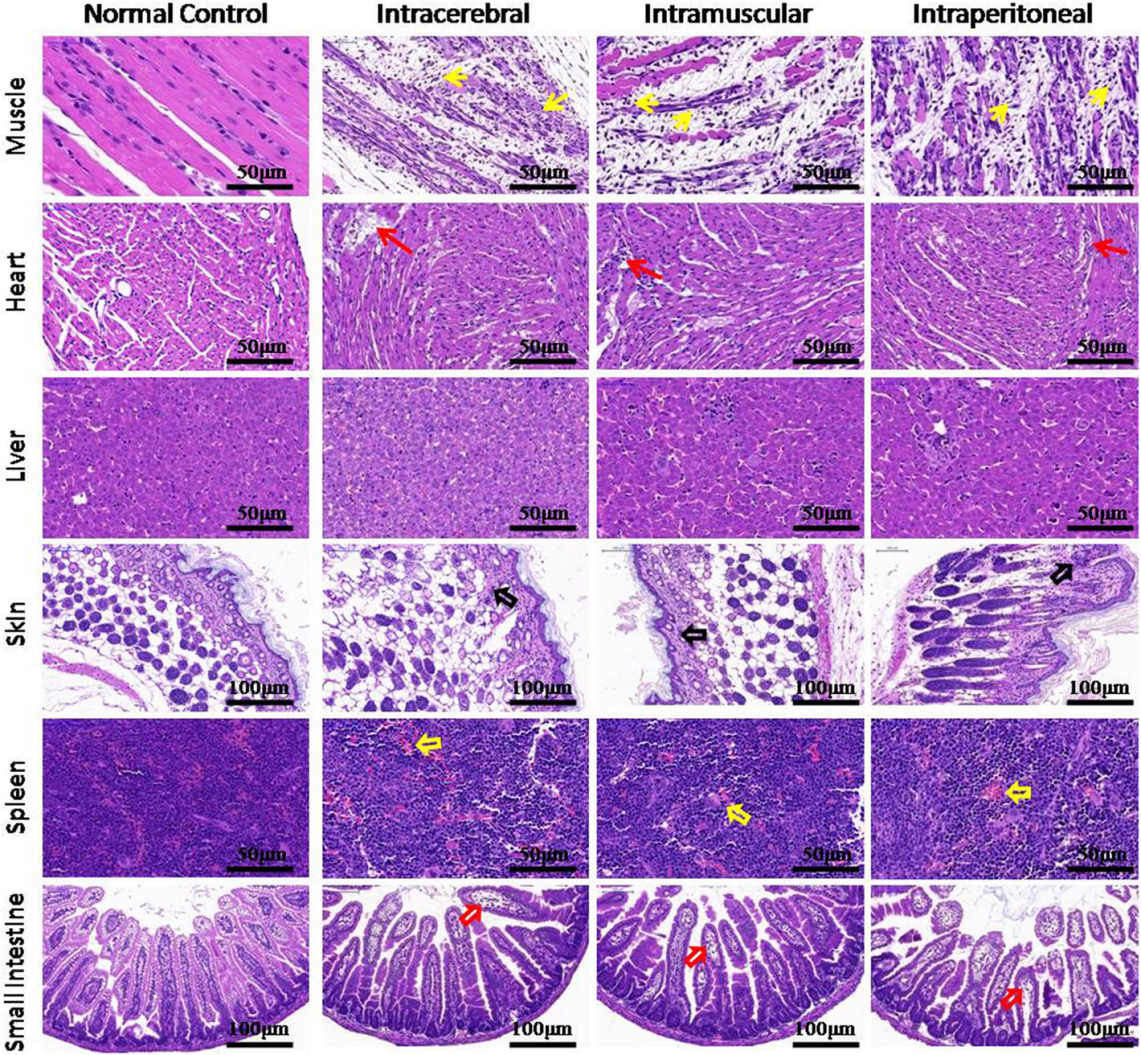
Representative histology of various tissues of ZZ1350-infected mice. Three-day-old BALB/c mice were intracerebral, intramuscular, intraperitoneal inoculated with ZZ1350 strain (2 × 10^6^ PFU/mouse), and histopathological changes of tissues from mice were examined at 7 dpi as described in Materials and Methods. Solid yellow arrows: severe necrotizing myositis with fragmentation of myofibers and inflammatory cells infiltration; Solid red arrows: myocarditis with inflammatory cells infiltration or cardiomyocytes necrosis; hollow black arrows: inflammatory cells infiltration; hollow yellow arrows: erythrocyte aggregation; hollow red arrows: villous atrophy with edema fluid accumulation in the lumen

Histopathological changes of skeletal muscle, hearts, livers, skin, spleens and small intestines were evaluated by H&E staining. As shown in Fig. 5, similarity lesions were observed in various mice tissues after any of three inoculation routes of ZZ1350 strain. Severe necrotizing myositis with fragmentation of myofibers and inflammatory cells infiltration (solid yellow arrows) were observed in skeletal muscle of infected mice. ZZ1350 infection caused myocarditis with inflammatory cells infiltration or cardiomyocytes necrosis (solid red arrows). Inflammatory cells infiltration (hollow black arrows), erythrocyte aggregation (hollow yellow arrows) and villous atrophy with edema fluid accumulation in the lumen (hollow red arrows) were observed in infected skin, spleens and small intestines respectively. However, we didn’t find any pronounced pathological changes in livers of infected mice.

### Pulmonary edema following CNS lesions induced by ZZ1350 infection

**Fig. 6:**
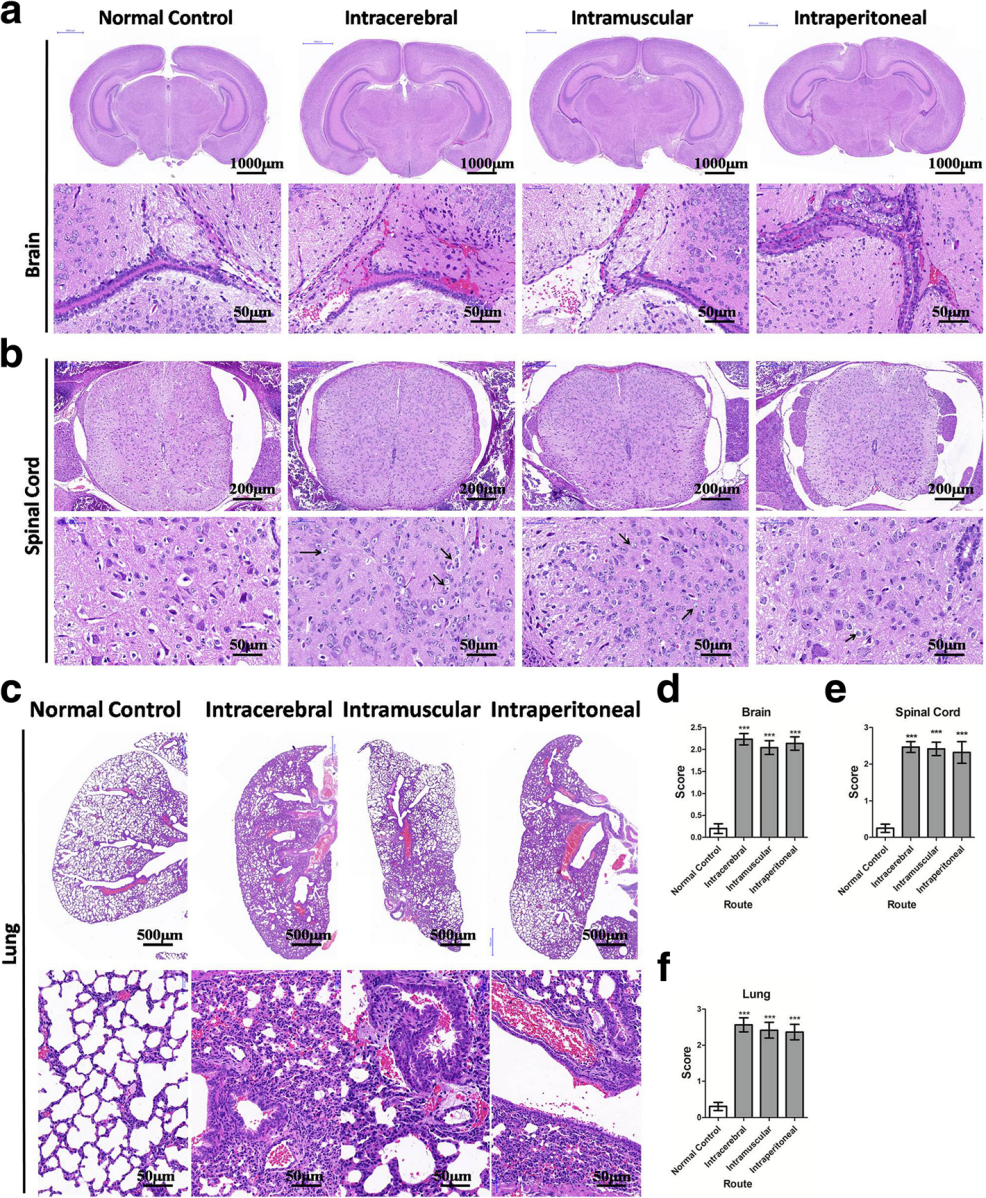
Histopathological analysis of CNS and lungs of mice. Histopathological changes of brain (**a**), spinal cord (**b**) and lung section (**c**) were evaluated by H&E staining. Histology score of brains (**d**), spinal cord (**e**) and lungs (**f**) was evaluated by a person blinded to the treatment groups. Data are expressed as mean ± SEM. *** *P* < 0.001, intracerebral (*n* = 15), intramuscular (*n* = 12), or intraperitoneal inoculation (*n* = 11) vs normal controls (*n* = 10)

Histopathological examinations of brains, spinal cord, lungs in mice with ZZ1350 infection were performed at 7 dpi. As shown in Fig. 6a, H&E stained brain tissues of infected animals with three inoculation routes presented focal minimal to slight peripheral erythrocyte exudation, relative to normal controls. GFAP is a biomarker of astrocytes which are associated with brain injury [12]. Compared to normal controls, a significant increase in the number of astrocytes (Additional file 5: Figure S4) was found in ZZ1350-infected mice (*P* < 0.001). Brain score (Fig. 6d) of infected animals with three inoculation routes was higher than that in normal controls (*P* < 0.001). Neuronal degeneration and loss (solid black arrows) were observed in the spinal cords of infected mice (Fig. 6b). Spinal cord score (Fig. 6e) of infected mice with three inoculation routes was higher than that in normal controls (*P* < 0.001). Histological and morphological alterations of lung tissues following ZZ1350 infection were evaluated by H&E staining, Masson’s Trichrome and PAS staining. A large number of red blood cells leakage around capillaries and large blood vessels was observed (Fig. 6c). Mucus secretion around bronchi was also found in lungs of infected animals by using Masson’s Trichrome and PAS staining (Additional file 6: Figure S5A). Alveolar space of the lungs was assessed by determination of L_m_ (Additional file 6: Figure S5B). Our result found that volume of alveolar representing as L_m_ from infected lungs was significantly increased, relative to normal controls (*P* < 0.001). Lung score (Fig. 6f) of infected mice with three inoculation routes was higher than that in normal controls (*P* < 0.001). These findings suggested that ZZ1350 infection could cause varying severity of CNS lesions, and even occurrence of pulmonary edema represented as red blood cells leakage, enhanced mucus production in bronchi and alveolar enlargement through three different inoculation routes.

### Association of CNS lesions with lung histological severity

**Fig. 7:**
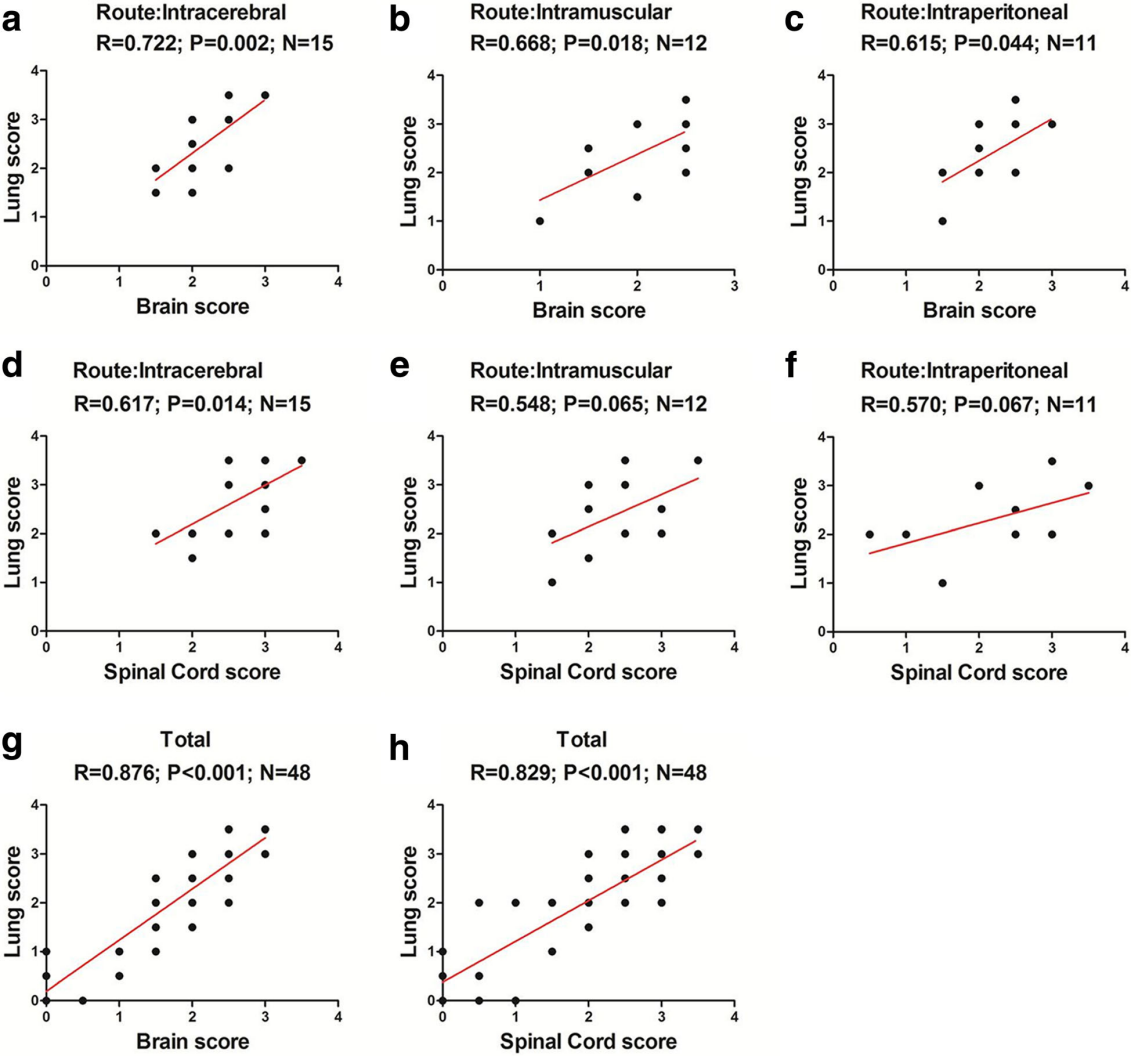
Association of CNS lesions with lung histological severity. Pearson’s correlation test was used to analyze association of brain score and spinal cord score with lung score of mice with intracerebral (**a** and **d**), intramuscular (**b** and **e**), intraperitoneal inoculation (**c** and **f**) and in total experimental animals (**g** and **h**)

As shown in Fig. 7. There were positive correlations between brain histology score and lung histology score of mice with intracerebral (*R* = 0.722, *P* = 0.002), intramuscular (*R* = 0.668, *P* = 0.018), intraperitoneal inoculation (*R* = 0.615, *P* = 0.044) and in total experimental animals (*R* = 0.876, *P* < 0.001). Additionally, There were also positive correlations between spinal cord histology score and lung histology score from mice with intracerebral inoculation (*R* = 0.617, *P* = 0.014) and in total experimental animals (*R* = 0.829, *P* < 0.001). These results suggested that CNS lesions caused by ZZ1350 infection was associated with occurrence of pulmonary edema.

## Discussion

EV71 infection has been a significant public health issue in the Asia-Pacific region, which can cause some adverse health effects in children under 5-year-old [3, 13–15]. So far, no effective treatment of drugs, vaccines and strategies has been proposed through clinical trials to prevent children death with severe symptoms [7, 16]. Generally, EV71-infected cases tend to have a faster disease progression, higher fever and a higher incidence of limb movement disorder, CNS lesions, pulmonary edema and death relative to other enterovirus strains [17]. However, the underlying mechanisms of EV71 infection-induced CNS lesions and pulmonary edema which increases risk of mortality remain largely unknown [5]. Here, we found an EV71 strain (ZZ1350) that could cause CNS lesions and pulmonary edema in BALB/c mice through three different inoculation routes. We also demonstrated that the occurrence of pulmonary edema was associated with CNS lesions after EV71 infection.

The EV71 strain ZZ1350, attributing to C4 subtype of virus, was isolated from a severe case in Zhengzhou Children’s Hospital, which exhibited a high cytotoxicity and fast replication in vitro experiments. Animal experiments have indicated that 3-day-old BALB/c mice is high susceptible to ZZ1350 and exhibit a various repertoire of clinical symptoms [5] ranging from skin lesions to paralysis, ataxia, tremors, acute encephalomyelitis and even respiratory disorders under intracerebral, intramuscular, intraperitoneal inoculation. Unlike other animal models [9, 18, 19], ZZ1350 was directly used to infect neonatal mice without pre-acclimated to mouse or mouse cell lines, making it more clinically relevant. Pathological changes were found in skeletal muscle, heart, skin, spleen and small intestine from infected mice. Skeletal muscle from mice with intracerebral, intramuscular, intraperitoneal inoculation exhibited the most serious lesion after infection regardless of the inoculation routes, which was consistent to the extent of virus invasion and replication in this section. These experimental results suggested that ZZ1350 strain was a muscle-tropic virus. Previous publications also reported that skeletal muscle was the most important site of EV71 replication [18, 20, 21]. It has been proposed that skeletal muscle supports persistent enterovirus infection and represents a viral source of entry into the CNS during poliovirus infection [22, 23]. This evidence is consistent with our finding of haemorrhagic lesions on the joint of limb. We also found viral replication in heart, liver and spleen. Consistent to other animal models [24–26], ZZ1350 infection causes myocarditis and spleen injury. Our finding supports clinical complications in children with HFMD suffering myocarditis and impaired immune function [2, 27]. Skin and small intestine lesions may explain hairless and weight loss after ZZ1350 infection.

CNS lesions are currently acknowledged as a trigger to cause paralysis, ataxia, tremors and encephalitis during human infection [28]. In clinical cases of EV71-associated fatal encephalomyelitis, viral antigens were mainly detected in neuronal cytoplasm and processes in the different brainstem nuclei and spinal cord [29]. Similarly, we also find viral replication in brain, spinal cord through VP1 expression and viral load determination regardless of inoculation routes. Based on above evidence, spinal cord may be a medium connecting brain and skeletal muscle that promotes EV71 virus spreading, which is also observed by Wei et al. [20]. Histopathological changes provide consistent results with viral invasion in the brain and spinal cord. Focal minimal to slight peripheral erythrocyte exudation and increasing number of astrocytes were found in infected brain section. Feng et al. reported that engulfment of EV71 by astrocytes facilitated viral replication and further aggravate brain lesions [30]. Neuronal degeneration and loss were found in anterior horns of spinal cords from ZZ1350-infected mice. As described in previous literatures [20, 31], inflammation and neuronal degeneration in anterior horns of the spinal cord are main injury induced by EV71 infection in human and mice. Taken together, our finding supports that the apparent limb paralysis after infection is of neurogenic origin and the infected neonatal mice presented neuropathological features which closely resemble those observed from clinical cases [29, 30].

Pulmonary edema is a main clinical complication causing death in severe cases [5, 32]. In our study, regardless of incoluation route, pulmonary pathologies including red blood cells leakage, increased alveolar volume, and mucus secretion around bronchi were observed. These results suggested ZZ1350 infection led to pulmonary edema with different degrees. Previous publications also reported pulmonary edema model in neonatal mice via intraperitoneal inoculation using mouse adapted strains [19, 21]. Hence, the occurrence of pulmonary edema after infection has nothing to do with inoculation routes. Large numbers of publications from animal experiments or clinical monitor indicated that the occurrence of pulmonary edema was of neurogenic origin due to CNS injury [19, 33, 34]. A controversial conclusion was reported that EV71 primarily replicated in skeletal muscle tissues, causing severe necrotizing myositis of respiratory-related muscles that further resulted in severe restrictive hypoventilation and subsequent hypoxia, which may explain the fatality of EV71-infected mice [35]. A study using rhesus monkeys showed that EV71 infection-induced CNS injury with intracerebral infection, was accompanied by clear inflammation of the lung [6]. Another study also indicated that inflammatory cytokines released in the brain facilitated pulmonary edema [36]. However, the convincing evidence about the relationship between CNS lesions and pulmonary disease has never been placed, and a consensus conclusion hasn’t been reached so far. Here, we scored the severity of brain, spinal cord and lung disorders and correlation analysis was applied. We found that brain score was positive correlated with lung score in total experimental mice and mice under three inoculation routes. At the same time, there were positive correlations between spinal cord score and lung score in total experimental mice and mice with intracerebral inoculation. Therefore, the extent of pulmonary disorders is associated with severity of CNS lesions induced by EV71 infection, which support the viewpoint from published literatures [6, 36].

## Conclusions

In summary, we isolate a clinical source EV71 strain (ZZ1350) and neonatal mice are very susceptible to the infection with this strain. We first uncover the association of CNS injury with pulmonary edema development in mice with three different inoculation routes using correlation analysis.

## Additional files

Additional file 1: Figure S1. RD cells exhibited obvious CPE following ZZ1350 infection. Control and infected RD cells at 60 hpi were captured under a light microscope (amplification: 100×). (TIFF 2294 kb)

Additional file 2: Sequence of the fragment of VP1 genome in ZZ1350 strain. (TXT 232 bytes)

Additional file 3: Figure S2. Weight loss after ZZ1350 infection. Body weight of mice (*n* = 7 for intracerebral inoculation; *n* = 6 for intramuscular inoculation; *n* = 6 for intraperitoneal inoculation and *n* = 5 for normal controls) was recorded every 2 days after ZZ1350 infection. Data are expressed as mean ± SEM. (TIFF 382 kb)

Additional file 4: Figure S3. ZZ1350 infection caused mice limb weakness. Percentage of limb paralysis of mice with three inoculation routes was recorded and calculated at 7 dpi. Limb paralysis (%) of mice with intracerebral (*n* = 7), intramuscular (*n* = 6) and intraperitoneal inoculation (*n* = 6) was 85.7%, 83.3%, 83.3% respectively. (TIFF 803 kb)

Additional file 5: Figure S4. ZZ1350 infection increased number of astrocytes in mouse brain. Astrocytes in brains from normal control (**A-a**) and mice with intracerebral (**A-b**), intramuscular (**A-c**), intraperitoneal inoculation (**A-d**) were determined by GFAP staining. **B**: Quantitative analysis of astrocytes number in slices of mice brains. Data are expressed as mean ± SEM. *** *P* < 0.001, intracerebral (*n* = 15), intramuscular (*n* = 12), or intraperitoneal inoculation (*n* = 11) vs normal controls (*n* = 10). (TIFF 3886 kb)

Additional file 6: Figure S5. ZZ1350 infection induced mucus production and alveolar space enlargement in mice lungs. Mucus production was determined by Masson’s Trichrome and PAS staining (**A**). L_m_ (**B**) was calculated based on 10 randomly selected fields in each section at 100× magnification with two crossed test lines. Data are expressed as mean ± SEM. *** *P* < 0.001, intracerebral (*n* = 15), intramuscular (*n* = 12), or intraperitoneal inoculation (*n* = 11) vs normal controls (*n* = 10). (TIFF 2944 kb)

## Abbreviations

CA16: Coxsackievirus A16
CNS: Central nervous system
CPE: Cytopathic effect
Dpi: Days post infection
EV: Enterovirus
FBS: Fetal bovine serum
GFAP: Glial fibrillary acidic protein
H&E: Haematoxylin and eosin
HFMD: Hand foot mouth disease
hpi: Hours post infection
IHC: Immunohistochemical
IVC: Individual ventilation cage
L_m_: Mean linear intercepts
PAS: Periodic Acid-Schiff
PBS: Phosphate-buffered saline
qRT-PCR: Real-time PCR
RD: Rhabdomyosarcoma
SEM: Standard error of the mean
Vero: African green monkey kidney
