# Correction to: Pulmonary edema following central nervous system lesions induced by a non-mouse-adapted EV71 strain in neonatal BALB/c mice

**DOI:** 10.1186/s12985-020-01480-1

**Published:** 2021-02-02

**Authors:** Yuefei Jin, Chao Zhang, Rongguang Zhang, Jingchao Ren, Shuaiyin Chen, Meili Sui, Guangyuan Zhou, Dejian Dang, Jiehui Zhu, Huifen Feng, Yuanlin Xi, Haiyan Yang, Guangcai Duan

**Affiliations:** 1 Department of Epidemiology, College of Public Health, Zhengzhou University, No. 100 Kexue Avenue, Zhengzhou, 450001 Henan China; 2 Henan Collaborative Innovation Center of Molecular Diagnosis and Laboratory Medicine, Xinxiang, People’s Republic of China; 3 Department of Epidemiology, College of Public Health, Xinxiang Medical University, Xinxiang, People’s Republic of China; 4 Department of Infectious Diseases, The Fifth Affiliated Hospital of Zhengzhou University, Zhengzhou, Henan People’s Republic of China

## Correction to: Virology Journal (2017) 14:243 https://doi.org/10.1186/s12985-017-0911-5

Following publication of the original article [1], the authors identified an unintended error in the Fig. 1c. The picture of EV71 (ZZ1350)-induced CPE in Vero cells at the MOI of 5.0 was the same for the picture of EV71 (ZZ1350)-induced CPE in Vero cells at the MOI of 1.0. These two pictures were captured under different backgrounds and were mistakenly put together during submission. In this Correction, the picture of EV71 (ZZ1350)-induced CPE in Vero cells at the MOI of 5.0 has now been replaced with the correct picture. We are sorry for the inconvenience caused by this unintended error. This correction does not affect the results and conclusions of this work.

**Figure 1:**
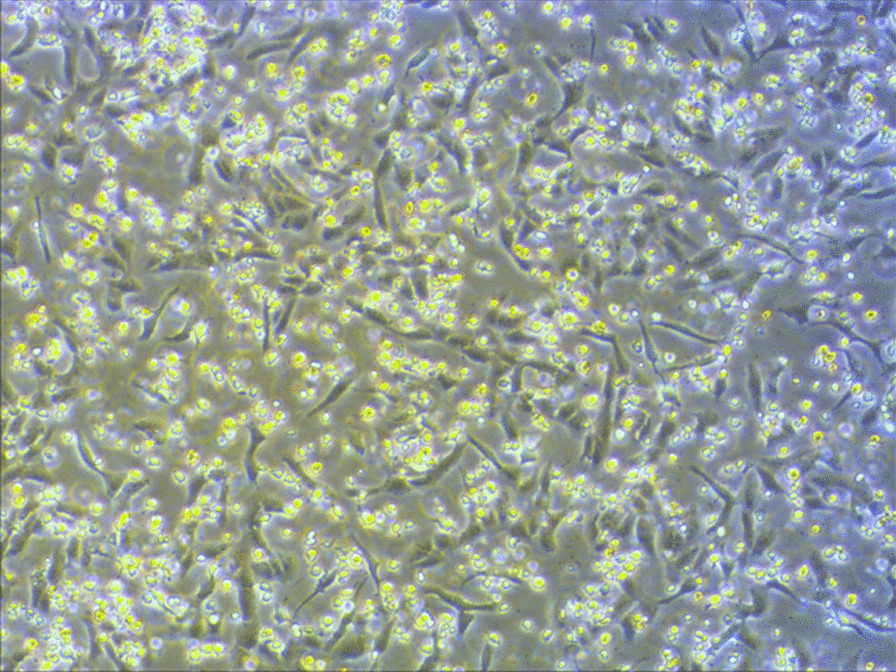

## References

[CR1] Jin X, et al. Virology Journal. 2017;14:243. 10.1186/s12985-017-0911-5.

