# Supplementary figures and images for: Pulmonary edema following central nervous system lesions induced by a non- mouse-adapted EV71 strain in neonatal BALB/c mice
Source: Virol J. 2017 Dec 28;14:243. doi: 10.1186/s12985-017-0911-5 (PMC5745784; doi:10.1186/s12985-017-0911-5)

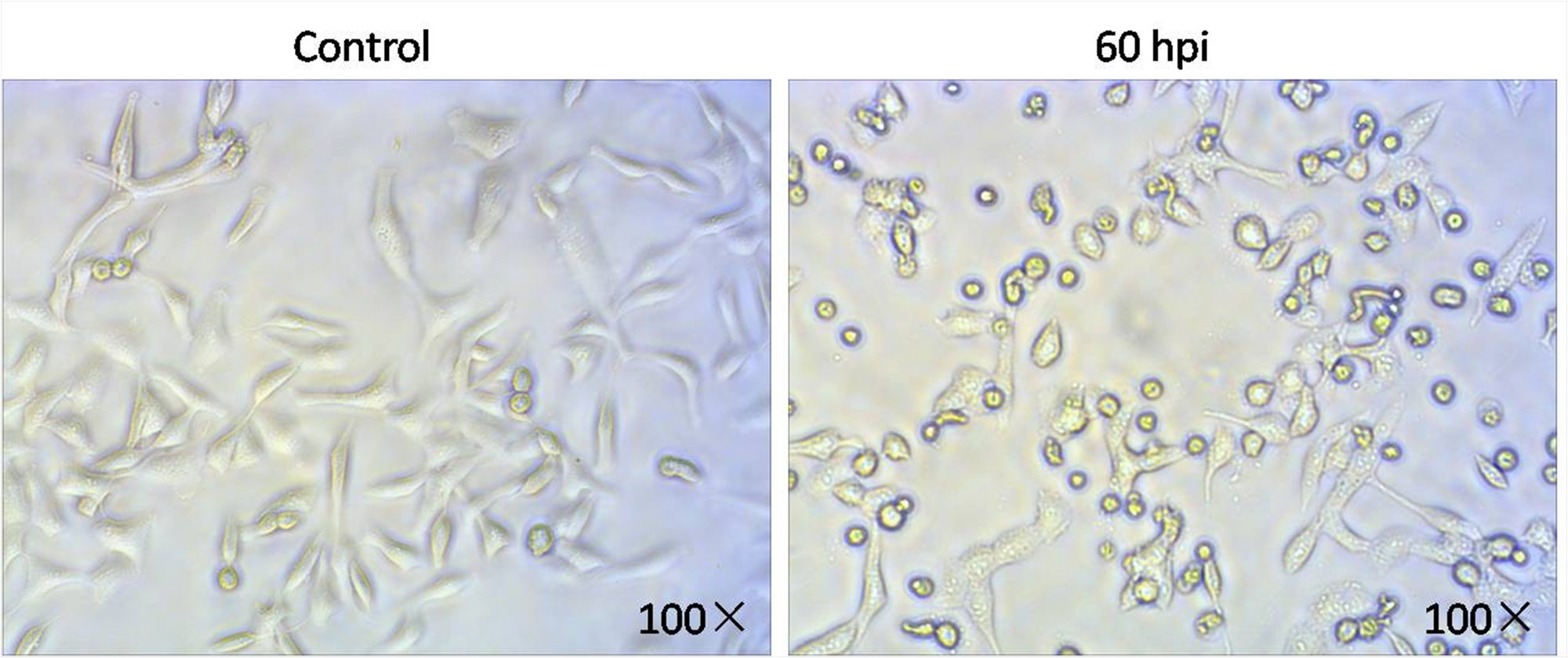

Supplement: Supplementary file 1 — RD cells exhibited obvious CPE following ZZ1350 infection. Control and infected RD cells at 60 hpi were captured under a light microscope (amplification: 100×). (TIFF 2294 kb) [file 12985_2017_911_MOESM1_ESM.tif]

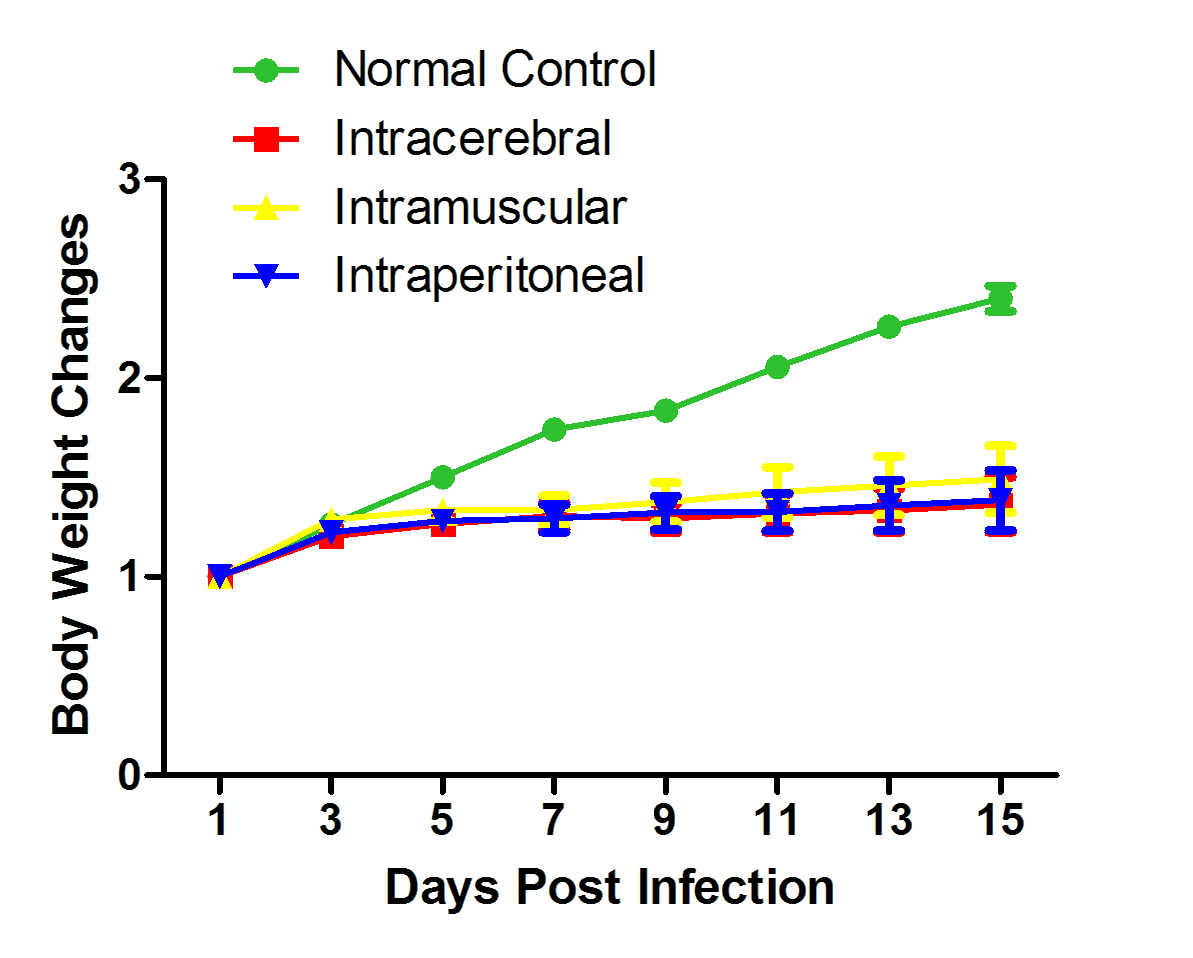

Supplement: Supplementary file 3 — Weight loss after ZZ1350 infection. Body weight of mice (n = 7 for intracerebral inoculation; n = 6 for intramuscular inoculation; n = 6 for intraperitoneal inoculation and n = 5 for normal controls) was recorded every 2 days after ZZ1350 infection. Data are expressed as mean ± SEM. (TIFF 382 kb) [file 12985_2017_911_MOESM3_ESM.tif]

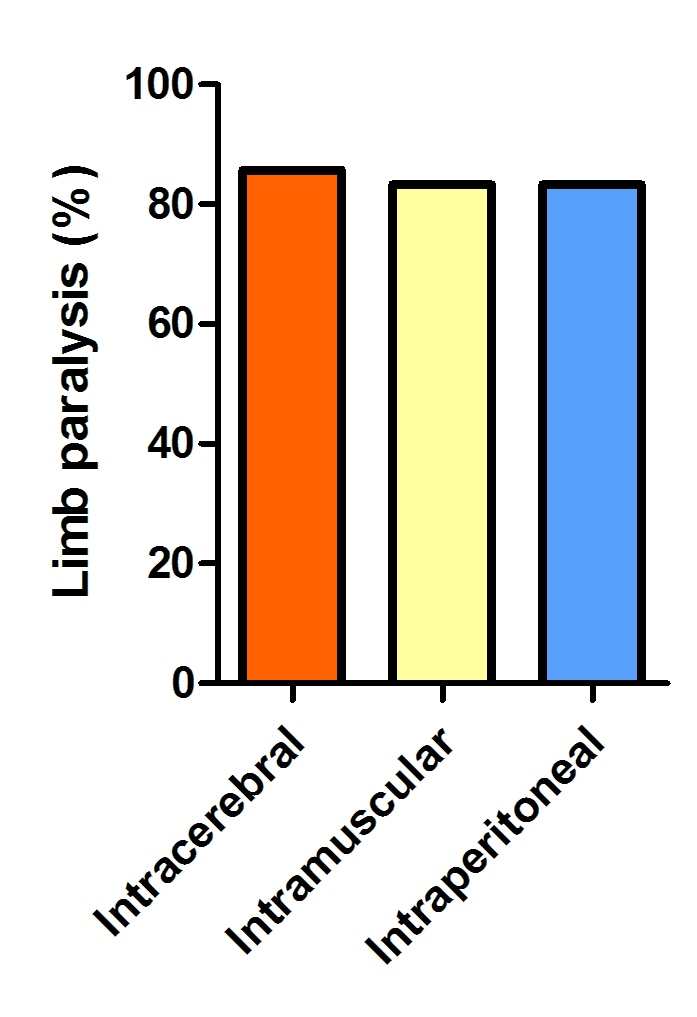

Supplement: Supplementary file 4 — ZZ1350 infection caused mice limb weakness. Percentage of limb paralysis of mice with three inoculation routes was recorded and calculated at 7 dpi. Limb paralysis (%) of mice with intracerebral (n = 7), intramuscular (n = 6) and intraperitoneal inoculation (n = 6) was 85.7%, 83.3%, 83.3% respectively. (TIFF 803 kb) [file 12985_2017_911_MOESM4_ESM.tif]

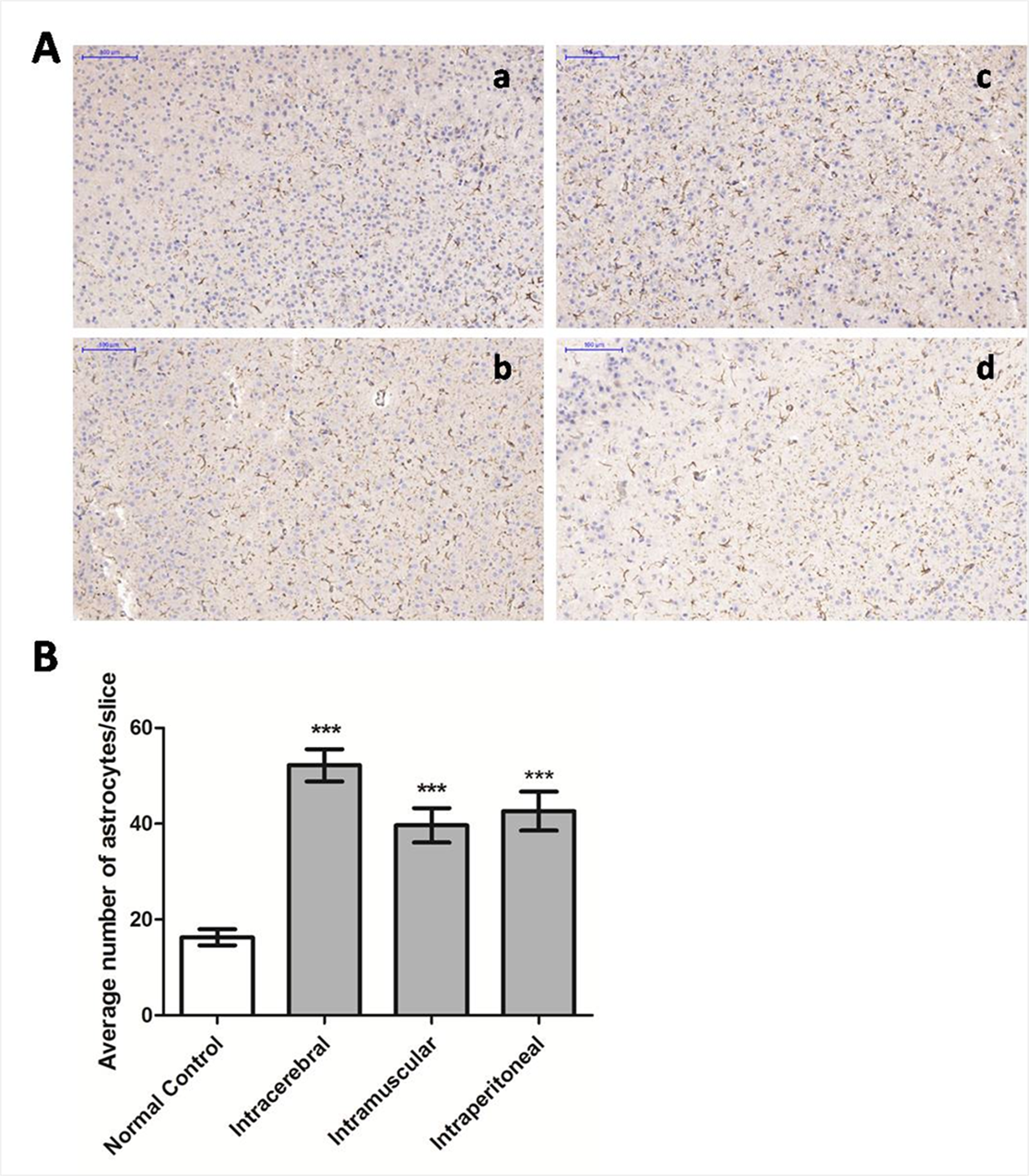

Supplement: Supplementary file 5 — ZZ1350 infection increased number of astrocytes in mouse brain. Astrocytes in brains from normal control (A-a) and mice with intracerebral (A-b), intramuscular (A-c), intraperitoneal inoculation (A-d) were determined by GFAP staining. B: Quantitative analysis of astrocytes number in slices of mice brains. Data are expressed as mean ± SEM. *** P < 0.001, intracerebral (n = 15), intramuscular (n = 12), or intraperitoneal inoculation (n = 11) vs normal controls (n = 10). (TIFF 3886 kb) [file 12985_2017_911_MOESM5_ESM.tif]

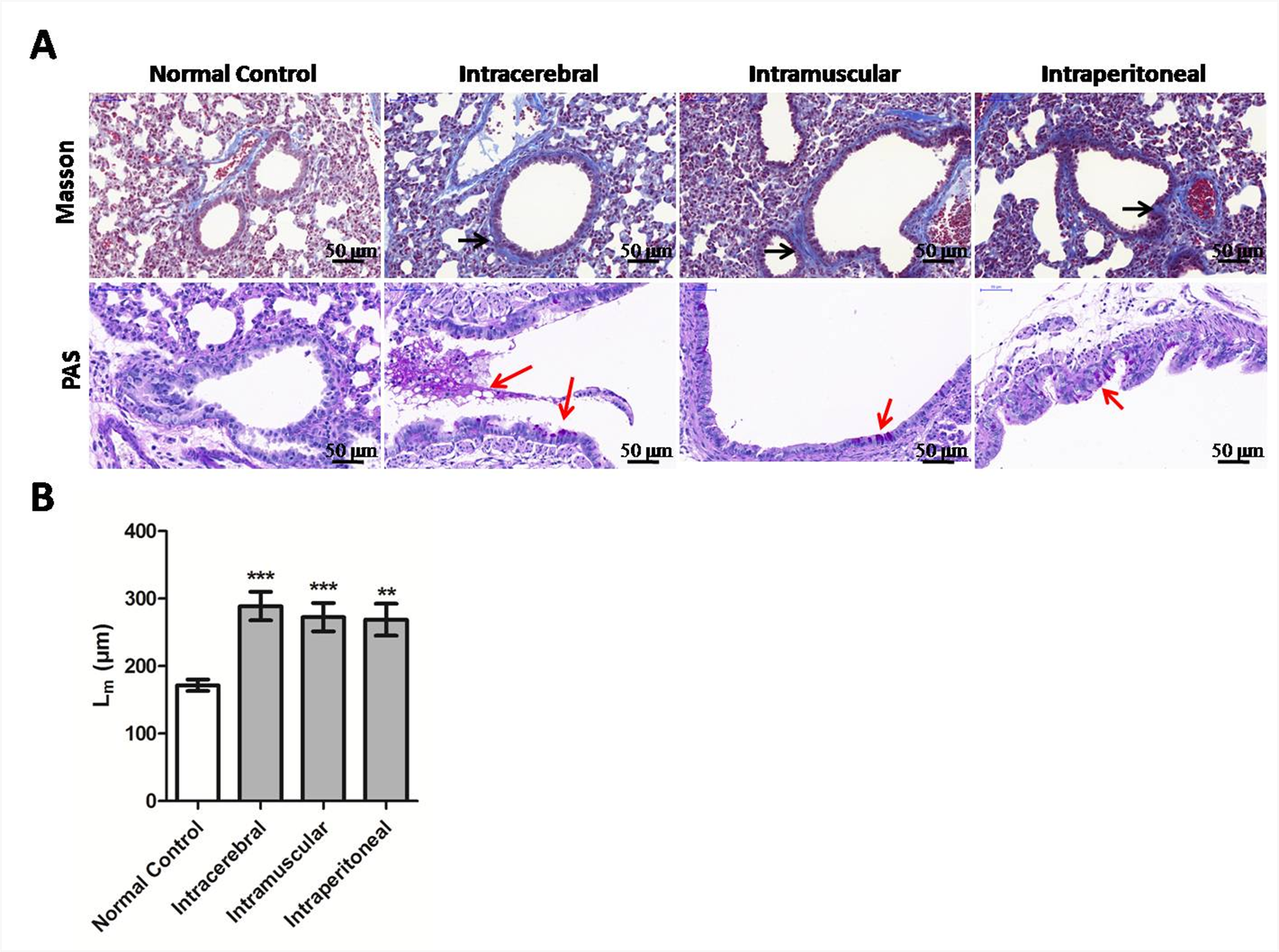

Supplement: Supplementary file 6 — ZZ1350 infection induced mucus production and alveolar space enlargement in mice lungs. Mucus production was determined by Masson’s Trichrome and PAS staining (A). Lm (B) was calculated based on 10 randomly selected fields in each section at 100× magnification with two crossed test lines. Data are expressed as mean ± SEM. *** P < 0.001, intracerebral (n = 15), intramuscular (n = 12), or intraperitoneal inoculation (n = 11) vs normal controls (n = 10). (TIFF 2944 kb) [file 12985_2017_911_MOESM6_ESM.tif]
